# Labeling of *Monilinia fructicola* with GFP and Its Validation for Studies on Host-Pathogen Interactions in Stone and Pome Fruit

**DOI:** 10.3390/genes10121033

**Published:** 2019-12-11

**Authors:** Silvia Rodríguez-Pires, Eduardo Antonio Espeso, Nuria Baró-Montel, Rosario Torres, Paloma Melgarejo, Antonieta De Cal

**Affiliations:** 1 Department of Plant Protection, Instituto Nacional de Investigación y Tecnología Agraria y Alimentaria, Ctra. de La Coruña Km. 7, 28040 Madrid, Spain; rpires.silvia@inia.es (S.R.-P.); melgar@inia.es (P.M.); 2 Department of Cellular and Molecular Biology, Centro de Investigaciones Biológicas, Consejo Superior de Investigaciones Científicas, Ramiro de Maeztu 9, 28040 Madrid, Spain; eespeso@cib.csic.es; 3 IRTA, XaRTA-Postharvest, Edifici Fruitcentre, Parc Científic i Tecnologic Agroalimentari de Lleida, 25003 Lleida, Spain; nuria.baro@irta.cat (N.B.-M.); rosario.torres@irta.cat (R.T.)

**Keywords:** *Agrobacterium*-mediated transformation, confocal microscopy, thermal asymmetric interlaced PCR (TAIL-PCR), brown rot

## Abstract

To compare *in vivo* the infection process of *Monilinia fructicola* on nectarines and apples using confocal microscopy it is necessary to transform a pathogenic strain with a construct expressing a fluorescent chromophore such as GFP. Thus, germinated conidia of the pathogen were transformed with *Agrobacterium tumefaciens* carrying the plasmid pPK2-hphgfp that allowed the expression of a fluorescent Hph-GFP chimera. The transformants were selected according to their resistance to hygromycin B, provided by the constitutive expression of the *hph*-*gfp* gene driven by the glyceraldehyde 3P dehydrogenase promoter of *Aspergillus nidulans*. The presence of T-DNA construct in the genomic DNA was confirmed by PCR using a range of specific primers. Subsequent PCR-mediated analyses proved integration of the transgene at a different genomic location in each transformant and the existence of structural reorganizations at these insertion points. The expression of Hph-GFP in three independent *M. fructicola* transformants was monitored by immunodetection and epifluorescence and confocal microscopy. The Atd9-*M. fructicola* transformant displayed no morphological defects and showed growth and pathogenic characteristics similar to the wild type. Microscopy analysis of the Atd9 transformant evidenced that nectarine infection by *M. fructicola* was at least three times faster than on apples.

## 1. Introduction

*Monilinia* spp. cause brown rot disease, one of the most important diseases on stone and pome fruit [1]. The distribution and prevalence of *Monilinia* spp. depend on the geographical area under study, the inoculum, and the host [2]. *M. fructicola* (G Winter) Honey and *M. laxa* (Aderhold & Ruhland) Honey are the main species causing brown rot in stone fruit currently in Spain, both with the same frequency after the first detection of *M. fructicola* in 2006 [3,4]. However, *M. fructigena* has been displacing as a minority species on stone fruit [4]. This displacement is supported by the effects of higher growth rate and aggressiveness factors in *M. fructicola* on stone fruit than *M. laxa* and *M. fructigena* [5]. Hyphal tubes from germinated *M. fructicola* conidia can penetrate the stone fruit surface through stomata or wounds and by degrading the cuticle of intact surface [1]. In nectarines, first brown-rot symptoms are visible 24 h post-inoculation on fruit surface and epidermal discoloration and brown spots are visible after 48 h [6]. This infection process involves initial cuticle decay, followed by the destruction of epidermal and mesocarp cells. The complete breakdown of cuticle and epidermis is the result of progressive vegetative growth and this infection process concludes with *M. fructicola* sporulation [6]. 

*M. fructicola* and *M. laxa* are species mostly found on stone fruit and only occasionally on pome fruit [2], or other fruit such as strawberry [7,8]. However, *M. fructigena* affects mainly apple and pear [9]. *M. fructicola* was first described as a pathogen on peaches in Spain in 2006 [3], while for the time being it has not been described as a pathogen on pome fruit in Spain; it is, however, present on pome fruit in other countries such as Czech Republic [10], Germany [11], Serbia [12], Italy [13], USA [14], China [15], and Brazil [16]. 

The higher *M. fructicola* prevalence on stone fruit than on pome fruit could be due to differences in the infection process described above. The green fluorescent protein (GFP) has proven useful in studies of host-pathogen interactions to detect and visualize the infection process in situ by confocal microscopy [17,18]. Although GFP-tagged fungal transformants have been obtained for a variety of postharvest pathogens [19,20,21], the use of this tool has not been extended to *M. fructicola* on stone or pome fruit. Only *Monilinia vaccinii-corymbosi* infections on blueberry flowers have been studied using fungal strains, which express GFP by transformation of their protoplasts [22]. Construction of a *M. fructicola* strain expressing GFP may facilitate the understanding of the less aggressive process of *M. fructicola* infection on pome fruit by confocal microscopy. However, the GFP insert could modify the fungal pathogenesis or alter any other type of genes essential for its growth. Therefore, once the mutant has been obtained, it is required to investigate where in the genome the construct expressing GFP was inserted and which activities might be affected. 

The aim of this study was to introduce the *hph*-*gfp* chimera into a pathogenic wild-type *M. fructicola* isolate using *Agrobacterium tumefaciens*-mediated transformation and resistance to hygromycin B as the selectable marker, as provided by the chimera. Purified Hph-GFP expressing transformants were characterized for maintaining their pathogenicity, germination, and growth capacity, compared to wild type strains. *M. fructicola* transformants were used to explore the infection process on nectarines and apples by confocal microscopy.

## 2. Materials and Methods

### 2.1. Fungi and Bacteria Growth Conditions 

*Monilinia fructicola* (wt38c) is a single spore isolate collected from a latent infection on a peach in Alfarrás (Lleida, Spain). Isolate wt38c showed high ability to infect stone fruit and produce conidia, and high resistance to carbendazim fungicide [23]. Wild type (wt38c) and transformant strains were stored as a conidial suspension in 20% glycerol at −80 °C for long-term storage, and as a culture on potato dextrose agar (PDA; Difco, Detroit, MI, USA) at 4 °C in darkness for short-term storage. PDA was supplemented with hygromycin B (100 µg/mL) for maintenance of transformants. 

**Figure 1:**
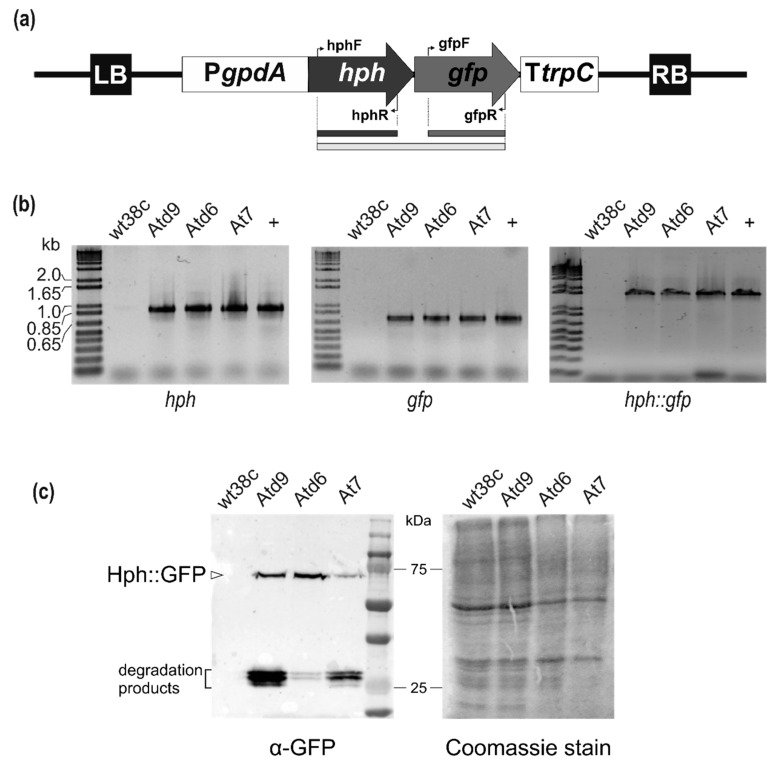
PCR and western blot analysis of transformants of *Monilinia fructicola*. (**a**) Schematic description of the T-DNA region of the pPK2hphgfp vector and primers locations. LB, left border; *PgpdA*, *Aspergillus nidulans* glyceraldehyde-3-phosphate dehydrogenase promoter; *hph*, hygromycin B phosphotransferase resistance gene; *gfp*, green fluorescent protein; *TtrpC*, *A. nidulans* tryptophan biosynthesis terminator; RB, right border. Primers for *hph* and *gfp* presence analysis are indicated by arrows. (**b**) PCR analysis of *M. fructicola* transformants, confirming the presence of *hph* gene (left), *gfp* gene (center) and the in-frame fusion of both genes (right). (**c**) Hph-GFP expression was confirmed by Western blot analysis of intracellular protein lysates from *M. fructicola* transformants using a specific primary antibody against GFP (left). Intracellular protein profiles of each transformant were verified with Coomassie staining and also served as loading control for western blot (right).

*Agrobacterium tumefaciens* strain LBA4404 carrying pPK2-hphgfp [24] provided by Dr. J. Martínez-Cruz (Microbiology Department, Malaga University, Spain) was used in fungal transformation experiments. The pPK2-hphgfp vector contains an in-frame fusion of the hygromycin B phosphotransferase resistance gene (*hph*) and green fluorescent protein (*gfp*) under control of *Aspergillus nidulans gpdA* promoter and *trpC* terminator (see Results section and Figure 1a). *A. tumefaciens* LBA4404 carrying pPK2-hphgfp was grown on LB agar containing 50 µg/mL rifampicin and 50 µg/mL kanamycin at 28 °C. 

### 2.2. Agrobacterium tumefaciens-Mediated M. fructicola Transformation 

A single colony of *A. tumefaciens* LBA4404 carrying pPK2-hphgfp was grown overnight in LB broth with rifampicin (50 µg/mL) and kanamycin (50 µg/mL) at 25 °C on an orbital shaker at 150 rpm. *A. tumefaciens* cells were collected by centrifugation and washed twice with induction medium (IM) as described by Michielse et al. [25]. The cell pellet was suspended in 5 mL of IM supplemented with acetosyringone (200 µM) and further cultivated for 4–5 h at 28 °C on an orbital shaker at 150 rpm. Next, *A. tumefaciens* culture was diluted to an OD_600_ of 0.3 in IM. Concurrently, conidia from 7-day-old *M. fructicola* (wt38c) colonies grown on PDA were harvested by scratching the surface of the plate and the spore suspension was adjusted to 1 × 10^6^ conidia mL^−1^ in IM. For transformation, this conidial suspension was incubated to promote germination of spores in IM at room temperature for 4 h at 150 rpm. Then, 100 µL of induced *A. tumefaciens* (OD_600_ of 0.3) were mixed with an equal volume of germinated conidia and spread on a 0.45 µm cellulose-nitrate membrane or cellophane sheets. The membranes or cellophane sheets were placed on IM with 1.5% agar and incubated at 22 °C for 2–3 days. After co-cultivation, membranes were transferred onto selective PDA plates supplemented with hygromycin B (100 µg/mL) and cefotaxime sodium salt (200 µM) to remove *Agrobacterium* cells. Hygromycin-resistant colonies of *M. fructicola* were selected individually and maintained on the selective medium PDA-H until further use.

### 2.3. Molecular Analyses of M. fructicola Transformants 

*M. fructicola* hygromycin-resistant transformants were grown for 3 days at 22 °C in potato dextrose broth (PDB) supplemented with hygromycin B (100 µg/mL). The mycelium was harvested by filtration through Miracloth (EMD Millipore Corporation, Billerica, MA, USA) and freeze-dried. Mycelium of potential transformants was ground to a fine powder using a FastPrep-24 (MP Biomedical, Solon, OH, USA; power 4, 20 sec/pulse) and genomic DNA was extracted by adding 1 mL of lysis solution (25 mM Tris-HCl pH 8.0, 0.25 M sucrose, 20 mM EDTA pH 8.0, 1% SDS), heating at 65 °C for 15 min, and then performing phenol-chloroform-isoamyl alcohol (25:24:1) extraction. Genomic DNA at the aqueous phase was precipitated using isopropanol, washed with 80% ethanol, and suspended in milli-Q water. To remove traces of RNA, DNA samples were treated with RNAseH for 1 h at 37 °C, then samples were stored at −20 °C until use. The presence of T-DNA in the genomic DNA of transformants was confirmed by PCR to detect the hygromycin resistance gene (primer pair hphF/hphR), *gfp* gene (primer pair gfpF/gfpR) (Table 1), and the fragment *hph*-*gfp* (primer pair hphF/gfpR) (see the Results section, Figure 1). 

Expression of GFP by transformants of *M. fructicola* was analyzed by western blot using total protein extracts according to Hernández-Ortiz and Espeso [26]. Hph-GFP fusion was detected using a polyclonal mouse anti-GFP (1/5000; Roche, Basel, Switzerland) as primary antibody. As the secondary antibody, a peroxidase-conjugated goat anti-mouse IgG immunoglobulin was used (1/4000; Jackson Immuno Research Laboratories, West Grove, PA, USA).

Thermal asymmetric interlaced PCR (TAIL-PCR) was used to identify the flanking regions to T-DNA. Genomic DNAs, extracted as described above, were used as templates in TAIL-PCR reactions. As an initial step, 15 cycles of single chain amplification were done using only the LB or RB primer. This ssDNA was used as a template for the first round of PCR, including LB/RB primers and the degenerate oligonucleotides (Table 1). The TAIL-PCR cycle setting conditions used were described previously [27] with several modifications (Table S1). Subsequently, second and third rounds were performed using 1 µl of the diluted product (1/10) of the previous PCR. After the third PCR reaction, products showing a reduction of 100 bp in size, due to the distance between LB2/LB3 and RB2/RB3, were excised from the agarose gel and purified using Nucleospin gel and PCR clean-up kits (Macherey-Nagel, Düren, Germany). Purified DNA fragments were sequenced (STAB VIDA, Caparica, Portugal) and compared to the available *M. fructicola* genome (NGKE01) using BLAST (NCBI, https://blast.ncbi.nlm.nih.gov/). Predictions of coding regions in the *M. fructicola* genome were done using AUGUSTUS [28] trained with *Botrytis cinerea* transcriptome. PCR results were compatible with a single integration event of TDNA in each transformant.

### 2.4. Characterization of GFP-M. fructicola Transformants

Morphology and colony growth were determined for *M. fructicola* transformants on PDA-H, where *M. fructicola* wild type (wt38c) on PDA was used as a control. Plugs (ø 5 mm) of actively growing mycelia were cut from the margins of a 7-day-old colony of each isolate and placed on the center of a sterile PDA-H or PDA Petri dish (ø 9 cm). The plates were then incubated for 14 days at 20–25 °C in the dark. The daily growth rate (mm day^−1^) was calculated from the individual measurements of colony diameter that were made on each day of the 14-day incubation using regression analysis. Three replicate plates were used per medium. Each assay was repeated at least twice. 

The aggressiveness of GFP-*M. fructicola* transformants was tested on disinfected wounded and unwounded peach fruit, cv. ‘Merryl O’Henry’. Each fruit was inoculated with 25 µl aqueous conidial suspension (10^4^ conidia ml^−1^) and incubated for 7 days in humidity (100%, RH) chambers at 20 °C. Each fruit was visually examined daily for symptoms of brown rot. The percentage of fruit with brown rot symptoms (%, brown rot incidence), the incubation period (the time interval between inoculation and the onset of symptoms), and latency period (the time interval between inoculation and the onset of sporulation) [5] were recorded for each infected fruit. Twenty fruits were used per type of inoculation point. The complete experiment was repeated twice. Data were statistically analyzed by one-way analysis of variance (ANOVA). When the results of the F-test were significant (*p* < 0.05), the means were compared using the LSD test (*p* < 0.05).

### 2.5. Microscopy Analysis of M. fructicola GFP-Transformants

Positive PCR transformants were analyzed for fluorescence emission. Mycelia samples of *M. fructicola* transformants grown on PDA-H were deposited onto a slide and fluorescence was imaged using a Leica DMRE microscope (Leica Microsystems, Wetzlar, Germany) with suitable filter system GFP ET, S (No: 11504174, Leica). Images were acquired using a Leica DFC550 camera driven by LAS v4.4.

Fruit infection process by GFP-*M. fructicola* transformants was visualized using a confocal laser scanning microscopy Leica SP2 (Leica Microsystems, Wetzlar, Germany). Nectarine (cv. Venus) and apple (cv. Golden Delicious) were surface disinfected [29] and dried in a laminar flow cabinet. Unwounded fruit surfaces were inoculated with 15 µL of 10^5^ conidia ml^−1^ of GFP-*M. fructicola* transformants with hygromycin B (100 µg/mL). Inoculated and unwounded nectarines were placed in individual sterilized boxes and incubated for 15 days at 20–25 °C in the darkness. Apples were placed in individual sterilized boxes and wounded with a sterilized scalpel 24 h after fungal inoculation. Wounded apples were incubated 14 days more at the same conditions. The infected tissue of each fruit was excised for the examination of surface and internal colonization at 0 h, 24 h, 5 days, and 15 days of fungal inoculation. Fluorescence of GFP was excited by a 488 nm laser and detected at 500–550 nm. When required, the fruit cell walls and membranes were stained using propidium iodide (10 µg/mL) and FM4-64 (17 µM) following [30,31]. Images were acquired using LAS X software v3.3.0.16799 (Leica Microsystems, Wetzlar, Germany) and further processed using ImageJ v1.48 (National Institutes of Health, Bethesda, MD, USA).

## 3. Results

### 3.1. Generating Green-Fluorescent M. fructicola Strains by Transformation

The growth of *M. fructicola* (wt38c), considered hereafter as a wild type strain, was completely inhibited on PDA supplemented with 100 µL/mL hygromycin B (PDA-H). This finding allowed a positive selection method for *Agrobacterium*-mediated transformation of T-DNA expressing the Hygromycin-B 4-O-kinase, Hph. The *A. tumefaciens* LBA4404 strain carries the pPK2-hphgfp plasmid, allowing the constitutive *gpdA*-driven expression of a Hph-GFP chimera, linking tolerance to hygromycin B and green fluorescence emission. Three putative *M. fructicola* transformants (Atd6, At7, and Atd9) were recovered on PDA-H medium. Atd9 was recovered on cellophane sheets, and At7 and Atd9 on cellulose-nitrate membrane. The stability of the genotypic modification was confirmed after at least five successive generations on PDA-H to all *M. fructicola* transformants. 

The presence of T-DNA in the genome of *M. fructicola* transformants was determined by PCR (Figure 1a). Combinations of oligonucleotide pairs were used to amplify either *hph* and *gfp* fragments or both. In all cases, genomic DNA from *M. fructicola* wild type (wt38c) was included as negative control. Detection of specific PCR products sizing, 877 bp for *hph* and 720 bp for *gfp*, as well as fusion of both genes *hph*-*gfp* 1632 bp (Figure 1b), confirmed the presence of T-DNA in the three transformants. Moreover, western blot analysis using antibodies against the GFP moiety confirmed the expression of the fusion protein Hph-GFP in protein extracts. The Hph-GFP chimera, with a predicted Mw of 64.9 kDa, was detected close to the 75 kDa marker in SDS-PAGE, as well as truncated forms comprising GFP (Mw: 26.9 kDa), which most likely correspond to degradation products of the chimera (Figure 1c).

**Figure 2:**
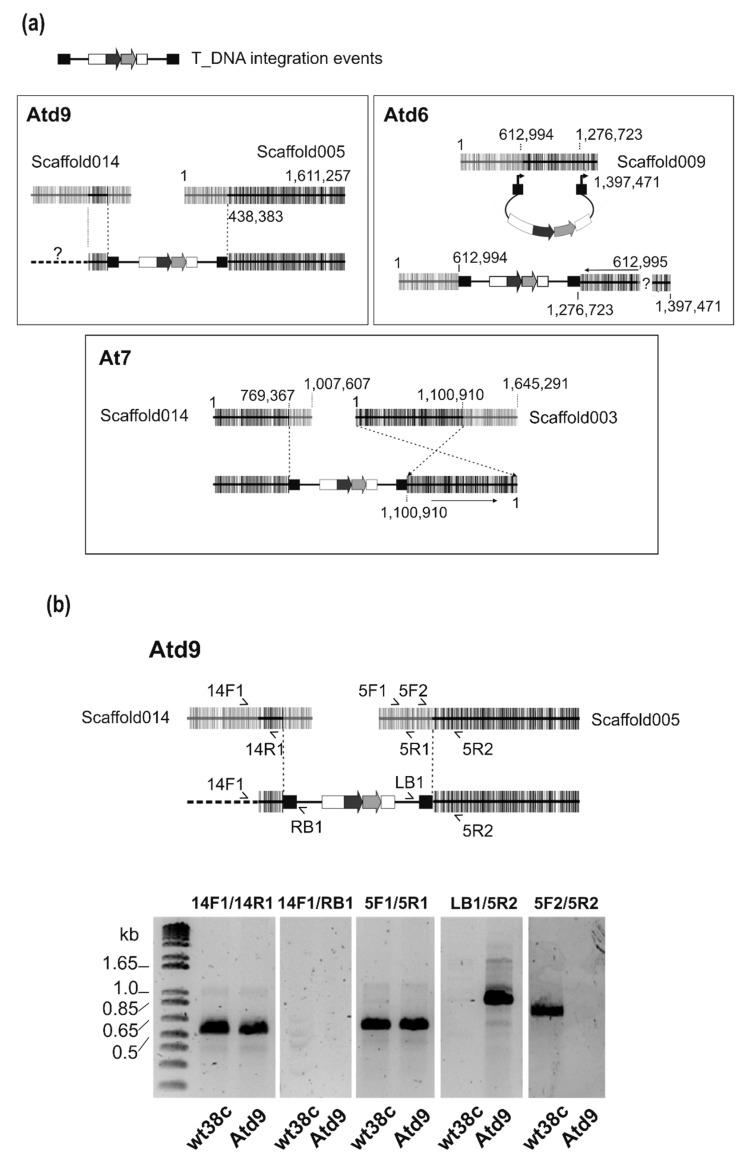
Identification of integration sites for T-DNA in transformants. Results from BLASTn searches of the NCBI database against *Monilinia fructicola* genome (LMK125, GenBank NGKE01). (**a**) Top, schematic representation of T-DNA. In boxes are interpretations of the integration events at the genomes of the three positive transformants. Unless indicated with an arrow, the scaffolds represent the DNA sequence orientation (5′–3′) and coordinates, as indicated in genome database. The dotted line in Atd9 corresponds to an unknown location, and dark vertical lines denote gene predictions. (**b**) Schematic representation of PCR analyses to identify the sequences flanking left and right borders of T-DNA and to verify the presence of remaining regions of scaffolds 014 and 005 in the genome of Atd9 transformant. Primers are listed in Table 1. At the bottom is shown the electrophoretic analysis of the PCR products.

Analyses of the flanking regions of the inserted T-DNAs were carried out by TAIL-PCR, demonstrating the random integration of T-DNAs into the *M. fructicola* genome. To identify the location of the construction insertion point in the genome of each transformant and recover the T-DNA border junctions, TAIL-PCR was performed (see experimental procedures, Table 1 for specific and degenerate oligonucleotides). The efficiency of TAIL-PCR was largely improved when several amplification cycles were included using only the specific LB1 or RB1 primer for each border of the construct. Primary amplification reactions, using LB1 and RB1 primers together with randomized primer, showed similar patterns of multiple bands (Figure S1). Secondary (LB2/RB2) and tertiary (LB3/RB3) nested PCRs (Figure S1) showed more specific amplifications of certain PCR products. Tertiary PCR products with a difference of approximately 100 bp from those obtained in the secondary PCRs were purified and sequenced. As a result, we identified 50–60 bp belonging to T-DNA flanking regions, followed by fungal genomic sequences (Table S2). BLAST searches of sequences matched with high similarity (97–99%) against the available *M. fructicola* genome (NGKE01). Notably, the flanking regions of the left and right borders of T-DNA for a given transformant were not always in the same scaffold (Figure 2—scaffolds for genomic assembly of LMK125 strain).

Five of six border junctions are located in intergenic regions and at the At7 right border within an intron of genic sequence. In transformant Atd6, we found the insertion of the left border at 1.2 kb upstream of the LMK125.3541 gene and the right border at 3.6 kb upstream of the LMK125.3699 gene, both into scaffold009. With respect to transformant At7, we found the insertion of the left border at 2 kb upstream of the LMK125.4967 gene in scaffold014 and the right border in the predicted coding region of LMK125.1494 gene, scaffold003. In transformant Atd9, we determined a sequence of 319 bp from scaffold014 at the left border, but of unknown location upstream from this point. However, we located the right border 250 bp upstream of LMK125.6942 gene in the scaffold005 (Figure 2a). 

Further PCR analysis of the Atd9 transformant showed the effect of T-DNA integration. This transformant with similar characteristics to the wild type carried a duplication of a 319 bp region from scaffold014 at the left border junction and genome reorganization of the right border. Firstly, we verified the integration location by PCR with a specific primer between genome prediction and T-DNA (14F1/LB1 and RB1/5R2). We recovered the right, but not the left border. Secondly, we checked the presence of the beginning of scaffold005 (5F2/5R2) and dismissed the possibility of deletion. Moreover, we proved the absence of amplification when primers located on both regions of the identified insertion point were used (5F2/5R2). Finally, 319 bp were recovered from scaffold014 in the left border, which was confirmed by PCR (14F1/14R1) and sequencing to be a duplication of this region (Figure 2b). 

### 3.2. Characterization of GFP-M. fructicola Transformants

**Figure 3:**
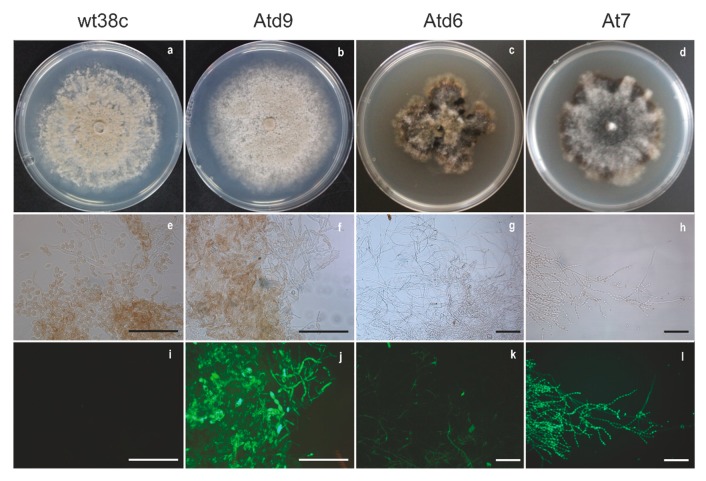
Colony growth of *M. fructicola* wild type (wt38c) on PDA (**a**) and GFP-*Monilinia fructicola* transformants (Atd9, Atd6, and Atd7) on hygromycin supplemented PDA (**b**–**d**). Conidia and hyphae of *M. fructicola* wild type (wt38c) on PDA (**e**,**i**) and GFP-*Monilinia fructicola* transformants (Atd9, Atd6, and At7) on hygromycin supplemented PDA (**f**–**h**,**j**–**l**) under optical microscopy (× 40) (Leica Microsystems, Wetzlar, Germany) with (**i**–**l**) or without (**e**–**h**) suitable filter system GFP ET, S (No: 11504174, Leica). Colonies of wt38c and Atd9 were incubated for 8 days at 22 °C, while Atd6 and At7 colonies were incubated for 14 days at the same temperature. Scale bars = 100 µm.

Two of the three GFP-*M. fructicola* transformants on PDA-H (Atd6 and At7) presented some phenotypic changes with respect to the wild type strain (wt38c) on PDA (Figure 3a,c,d). Growth rates of Atd6 (3.9 mm day^−1^) and At7 (4.4 mm day^−1^) transformants were significantly lower than wt38c (9.2 mm day^−1^). Colony color, stromata, sporulation, and concentric growth circles were also significantly different among wt38c and Atd6 or At7 (Figure 3e,g,h). However, no difference, neither of colony and sporulation appearance nor of growth rate, were observed between the wild strain (wt38c) on PDA and Atd9-*M. fructicola* transformant on PDA-H (Figure 3a,b,e,f).

**Figure 4:**
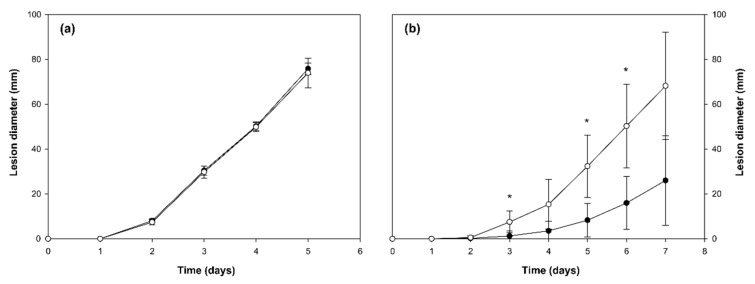
Comparison of *Monilinia fructicola* wild type wt38c (●) and Atd9-*M. fructicola* transformant (◌) growth rate (mm) in wounded (**a**) and non-wounded (**b**) ‘Merryl O’Henry’ peaches. Wounded fruit were inoculated with 10 µl of each strain at 10^4^ (100 conidia per inoculation site) and non-wounded fruit were inoculated with 10 µl at 10^5^ conidia ml^−1^ (1000 conidia per inoculation site) and incubated for 5 and 7 days, respectively, at 20 °C and 100% relative humidity. Each point represents the mean and vertical bars indicate the standard deviation of the mean (n = 40). For each point, asterisks denote significant differences between strains according to analysis of variance (ANOVA) and LSD test (*p* < 0.05).

Aggressiveness tests were only carried out with the At9-*M. fructicola* transformant and wild type wt38c on disinfected wounded and unwounded peaches. Brown rot incidence caused by Atd9-*M. fructicola* transformant on wounded peaches was similar to that caused by the wild type (wt38c) (Figure 4a). Only significant differences were observed between Atd9-*M. fructicola* transformant and the wild type wt38c on unwounded peaches after five days of incubation at 20 °C and 100% RH (Figure 4b). 

### 3.3. Microscopy Analysis of M. fructicola GFP-Transformants

Green autofluorescence background was not observed in cells of the wild type strain (wt38c) (Figure 3i). However, the three recovered GFP-*M. fructicola* transformants (Atd6, At7, and Atd9) showed GFP fluorescence when they grew on PDA-H (Figure 3j–l), although Atd6 displayed an irregular fluorescence distribution throughout its mycelia (Figure 3k). 

**Figure 5:**
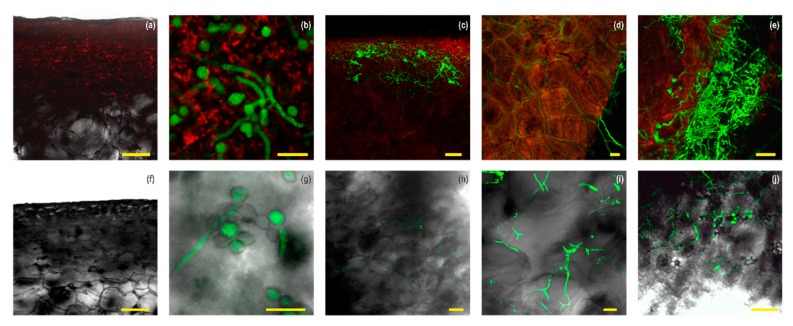
Infection process by Atd9-*M. fructicola* transformant on nectarines (**a**–**e**) and apples (**f**–**j**) under confocal laser scanning microscopy Leica SP2 (Leica Microsystems, Wetzlar, Germany) for 14 days of incubation at 22 °C. Nectarine’s tissues were stained with propidium iodide and FM4-64 (**a**–**e**) and apple tissues were visualized without stain (**f**–**j**). Atd9-*M. fructicola* transformant conidia on nectarine (**a**) and apple (**f**) surface show recent inoculation by the pathogen. Scale bars = 100 µm. Conidia and germ tubes on nectarine (**b**) and apple (**g**) surface after 1 day of pathogen inoculation. Scale bars = 25 µm. Colonization of the uppermost layer of epidermal cells in nectarine (**c**,**d**; the latter is a magnification of experiment shown in **c**) and in apple (**h** and **i**, the latter is a magnification of experiment shown in h) by Atd9-*M. fructicola* hyphae after 4 or 14 days of pathogen inoculation, respectively. Scale bars = 75 µm (**c**,**h**) and 50 µm (**d**,**i**). Colonization and sporulation on nectarine (**e**) and apple (**j**) surface, after 4 or 14 days of Atd9-*M. fructicola* inoculation, respectively. Scale bars = 50 µm. All images represent maximum projections of a z-stack.

Atd9-*M. fructicola* transformant infection process on nectarines and apples at 22 °C was studied using confocal microscopy (Figure 5). Visible epidermal discoloration and brown spots were only observed at the inoculum sites on unwounded nectarine surfaces after 24 h incubation, while disease signs were not evident on apple fruit. Confocal microscopy showed that a higher conidial germination of Atd9 could be observed on surfaces of unwounded nectarines than on wounded apples after 24 h of pathogen inoculation (Figure 5b,g).

Notably, four days after inoculation, massive mesocarp colonization and profuse external sporulation by Atd9-*M. fructicola* were visible on unwounded nectarine (Figure 5c–e). Atd9 was spread into the apoplastic nectarine space (Figure 5e) through thin and branched hyphae. Thick hyphal groups were observed in certain intracellular nectarine spaces four days after inoculation. In contrast, brown rot symptoms and Atd9-*M. fructicola* transformant colonization was only found in the uppermost layer of epidermal cells in wounded apple after 14 days of pathogen inoculation (Figure 5h,i). 

## 4. Discussion

The use of an aggressive *M. fructicola* transformant that expresses a green fluorescent chimera has allowed comparison of the infection process on unwounded nectarine against that on wounded apple over a 15-day incubation period. The *M. fructicola* infection process for nectarines observed by confocal microscopy in the present study was similar to that reported by García-Benítez et al. [6] by optical microscopy using infected tissue histological cuts, which required a great deal of time and effort to obtain. Confocal microscopy is a potent technique that allows in depth high-resolution analysis from many samples [32]. Thus, it allowed us to quickly study the infection and colonization on different hosts by GFP-Monilinia. Fluorescent-tagged proteins have been successfully used in living tissues to study host-pathogen interactions [17,33]. Other plant-pathogen interactions such as *Botrytis cinerea* in strawberry [20], *Fusarium oxysporum* in tomato [34] or *Penicillium digitatum* in orange [35] have been reported, where the expression of a fluorescent protein did not affect their pathogenic capacity. 

GFP-*M. fructicola* transformant germinated on the surface of nectarines and apples, but apple colonization by the pathogen was notably delayed compared with nectarines. This observation is in agreement with *M. fructicola* being classified as a secondary pathogen on apples, while it is a prominent pathogen on stone fruit [1]. *M. fructicola*, *M. fructigena*, and *M. laxa* are polytrophs and infect a wide range of Rosaceae species, but there is host specialization in some cases [36]. *M. fructicola* is mainly limited to fruit and blossoms in stone fruit orchards (peach, nectarine, plum, and apricots). However, *M. fructicola* could affect apples at or near maturity with tan to white zones of sporulation [2,11,16].

We have transformed germinated conidia of *M. fructicola* using *Agrobacterium tumefaciens* and expressed a chimera consisting of GFP fused to the protein conferring HygB resistance, Hph. Other authors have reported transformed *M. fructicola* isolates (Bmpc-7, DL25W and MUK-1) to express genes of interest under control of native promoters [37,38,39,40] and also with *A. nidulans trpC* as an exogenous promoter [41,42]. A preliminary *Agrobacterium tumefaciens*-mediated transformation of *M. fructicola* with a binary vector expressing GFP has been reported [43]. However, no GFP-expressing *M. fructicola* strains have been used on pathogenicity works at the moment [38,39,41,43]. This is the first report describing the use of a *M. fructicola* transformant expressing GFP in comparison of the interaction of this pathogen with nectarine and apple using confocal microscopy.

In this work, three GFP-*M. fructicola* transformants, confirmed by PCR and western blotting, were recovered using ATM-transformation and germinated conidia. Only transformant Atd9 showed similar growth and pathogenic characteristics to wild type, but a slightly increased virulence on unwounded nectarines was also observed. The presence of morphological and pathogenic defects suggested T-DNA integration effect during the transformation process. Thus, we used a standardized technique to verify the insertion limits of T-DNA integration. The initial 50–60 bp from recovery sequences belong to the pPK2-hphgfp vector and the following sequence aligns with *M. fructicola* genomes, confirming that genome integration and three independent transformation events have occurred due to their different location. In model plant-pathogen *Magnaporthe oryzae*, two systematic analyses of T-DNA insertion revealed that most transformation events do not cause extreme chromosomal rearrangements, although they produced deletions, inversions, and translocations [44,45,46]. We observed that in Atd6 (referenced to the *M. fructicola* LMK125 genome), an inversion of part of the scaffold might have been generated. ATMT showed integration bias toward the non-coding region and promoter regions in *M. oryzae* and *L. maculans* [44,45,46,47]. Considering the automatic prediction of genes, in the right border of At7, the ATMT transformation seems to have disrupted a gene. Atd6 and At7 transformants presented a different phenotype to the wild type strain, which can be explained in part by a T-DNA integration effect. PCR analyses of Atd9 transformant suggested that most nuclei carry the construct although we cannot discard the presence of wild type nuclei in the mycelium. In addition, these analyses showed that the integration of the construct resulted in a duplication of a small genomic region in the left border. The broader PCR analysis (Figure 2b) detected regions adjacent to the integration sites in scaffolds 014 and 005, supporting the idea of a reorganization of the original genome of *M. fructicola* wt38c in the Atd9 transformant. The T-DNA integration effects described for the three transformants may be, in part, the cause of the low number of *M. fructicola* transformants recovered from ATMT. Here, we used the constitutive glyceraldehyde-3-phosphate dehydrogenase promoter (*gpdA*) from *A. nidulans* to drive the expression of the *gfp* reporter gene fused with the hygromycin B resistance gene. As reporter for other fungal species, the *gpdA* promoter from *A. nidulans* is an efficient genetic tool to constitutively express genes of interest [26,48]. 

## 5. Conclusions

GFP-*M. fructicola* has allowed to demonstrate certain host specificity by the pathogen. Furthermore, Atd9-*M. fructicola* had similar properties as the wild type, hence opening a new avenue for future modifications in *M. fructicola* dedicated to deciphering its pathogenic mechanisms in different fruit stages and host compatibility. 

## Figures and Tables

**Table 1:** Primers used in this study.

Primer	Sequence (5′–3′)	Description	Reference
gfpF	CCCATGAGTAAAGGAGAAGAACTT	*gfp* gene	[18]
gfpR	CTATTTGTATAGTTCATCCATGCCATGTGTA		
hphF	TTCGATGTAGGAGGGCGTGGATATG	*hph* gene	[18]
hphR	GGTTTCCACTATCGGCGAGTACTTC		
LB1	TTAATTGCGTTGCGCTCACTGC	TAIL-PCR	[18]
LB2	GCTTTCCAGTCGGGAAACCTGTC		
LB3	GAGCAATTCGGCGTTAATTCAGT		
RB1	GGCACTGGCCGTCGTTTTACAAC		
RB2	AACGTCGTGACTGGGAAAACCCT		
RB3	CCCTTCCCAACAGTTGCGCA		
AD1	WGTGNAGWANCANAGA		
AD2	NGTCGASWGANAWGAA		
InsLB (Atd9)	AATACCCTGTTGAGATTTGG	Left/right border junction of Atd9 strain	This study
InsRB (Atd9)	TACTGGCTAGGCAAGAGTCTGG		
scf005-F	TCATCATAACGGCAGGGAGG	Amplification of putative deletion on Atd9 strain	This study
scf005-R	GCCACCACAAATTCCAAGCG		
scf014-F	TGTGGCGTCTGCTTGTATCC	Amplification of putative duplication on Atd9 strain	This study
sc014-R	TTTGGCGAGGTCATCATAGC		

## Supplementary Materials

The following are available online at https://www.mdpi.com/2073-4425/10/12/1033/s1, Figure S1: TAIL-PCR analysis of border junctions of *M. fructicola* transformants. The agarose gel analysis from top to the bottom show products of three rounds of PCR with specific primer for the left (LB1/LB2/LB3) or right (RB1/RB2/RB3) border of T-DNA and degenerate oligonucleotides (AD1/AD2) of each *M. fructicola* transformant (Atd9, Atd6 and At7). The bands surrounded by dashed squares were excised from the gel and sequenced, Table S1: Conditions used for TAIL-PCR; Table S2. Sequence analysis of the T-DNA border junctions (left and right borders) in the *Monilinia fructicola* transformants (Atd9, Atd6, and AT7).
