# Supplementary material for: Labeling of Monilinia fructicola with GFP and Its Validation for Studies on Host-Pathogen Interactions in Stone and Pome Fruit
Source: Genes (Basel). 2019 Dec 11;10(12):1033. doi: 10.3390/genes10121033 (PMC6947648; doi:10.3390/genes10121033)
Supplement: Supplementary file 1 [file genes-10-01033-s001.zip › genes-642786- Figure S1.pdf]

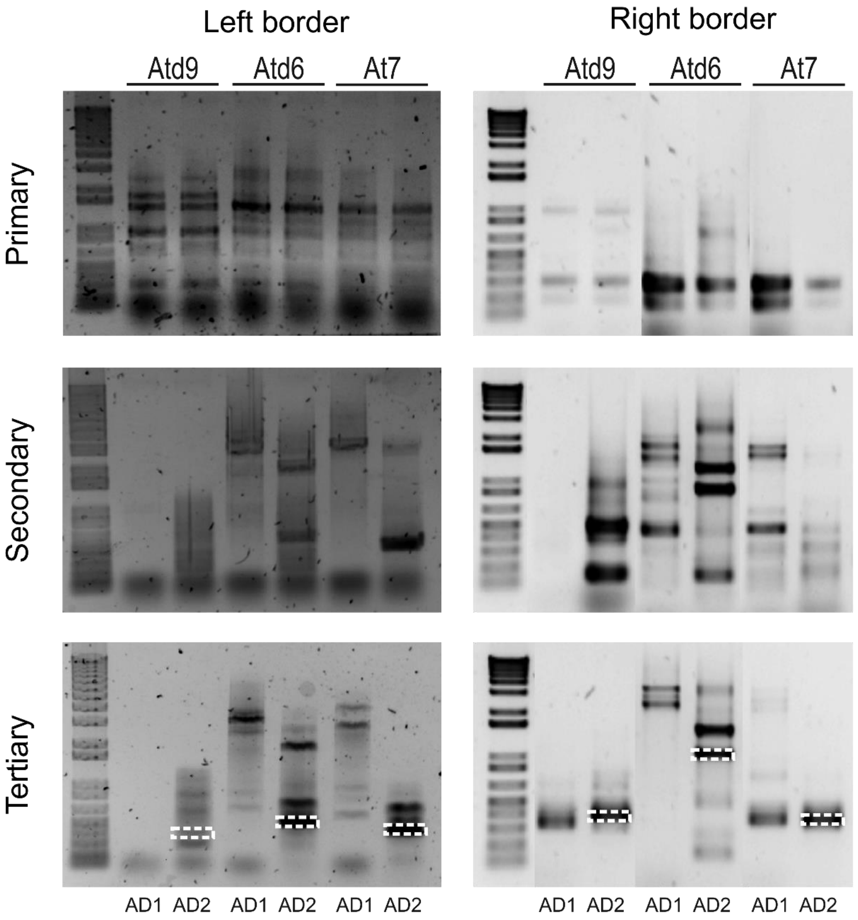

Figure S1. TAIL-PCR analysis of border junctions of *M. fructicola* transformants. The agarose gel analysis from top to the bottom show products of three rounds of PCR with specific primer for the left (LB1/LB2/LB3) or right (RB1/RB2/RB3) border of T-DNA and degenerate oligonucleotides (AD1/AD2) of each *M. fructicola* transformant (Atd9, Atd6 and At7). The bands surrounded by dashed squares were excised from the gel and sequenced.
