# Supplementary material for: Labeling of Monilinia fructicola with GFP and Its Validation for Studies on Host-Pathogen Interactions in Stone and Pome Fruit
Source: Genes (Basel). 2019 Dec 11;10(12):1033. doi: 10.3390/genes10121033 (PMC6947648; doi:10.3390/genes10121033)
Supplement: Supplementary file 1 [file genes-10-01033-s001.zip › genes-642786- Table S1.pdf]

Table S1. Conditions used for TAIL-PCR.

Reaction	Number of Cycles	Thermal settings
PreTAIL-PCR	x1	94°C 2 min
	x15	94°C 30 s, 58°C 30 s, 72°C 1 min
Primary	x1	92°C 3 min, 95°C 2min
	x5	94°C 30 s, 64°C 1min, 72°C 2min
	x1	94°C 30 s, 30°C ramping to 72°C in 3 min
	x1	72°C 2 min
	x10	94°C 30 s, 44°C 1min, 72°C 2min
	x15	94°C 30 s, 66°C 1min, 72°C 2min
	x15	94°C 30 s, 64°C 1min, 72°C 2min
	x15	94°C 30 s, 44°C 1min, 72°C 2min
	x1	72°C 5 min
Secondary	x1	94°C 2 min
	x30	94°C 10 s, 60°C 1min, 72°C 2min
	x15	94°C 10 s, 44°C 1min, 72°C 2min
	x1	72°C 5 min
Tertiary	x1	94°C 2 min
	x30	94°C 30 s, 42°C 1min, 72°C 2min
	x1	72°C 5 min
