# Supplementary material for: Labeling of Monilinia fructicola with GFP and Its Validation for Studies on Host-Pathogen Interactions in Stone and Pome Fruit
Source: Genes (Basel). 2019 Dec 11;10(12):1033. doi: 10.3390/genes10121033 (PMC6947648; doi:10.3390/genes10121033)
Supplement: Supplementary file 1 [file genes-10-01033-s001.zip › genes-642786- Table S2.pdf]

Table S2. Sequence analysis of the T-DNA border junctions (left and right borders) in the *Monilinia fructicola* transformants (Atd9, Atd6, and AT7)

	LEFT BORDER	RIGHT BORDER
Atd9<br>(38cGFP)	gacagtctagtttaaccagttgcaagatctaccgcagcccgttaagacatctcattacgcgcacgagcaga<br>aggaaaggcaaatcgaccccgtacaaagaaaaaaacctgtccgagccgctgcactctcctcgtcgagctt<br>aggttcgataaat**C**g**ATATAACACCACATTTGTTTAACTGCGAATCTGTT**<br>**GAATTATTGTGT…**	**…TTTCCCGCCTTCAGTTTAAACTATCAGTGTTTG**tgtcgtag<br>agtagctgagcaagaagggcgccaggattcgggaccaagtggttttggaaatccgtatag<br>ctgcgtcagtttgggttgtcccgcattcacatattatatatatggaggaatatccccagtggag<br>ggcacctcaagaccttgttgtaacagaacttcagatcctccaa
Atd6	gacgaaaactgtattttctataacttggaaccaacagtggccctaaccaaaaggtctaaccaaaaggttccg<br>g<br>cccccttagttattgacggatagagatggatcgtgaagtaaaggggagtgtatgggcacatacaaatgg<br>**GTCCTATATAACACCACATTTGTTTAACTGCGAATCTGTTG-**<br>**ATTATTGTGT…**	**…TTTCCCGCCTTCAGTTTAAACTATCAGTGTTTGA**g**A**aaa<br>atagaccagatagtgaaatggttctcggcgaagattgttccaccgacaaggcagctttctttt<br>caacagacccttgcggccctgcatataagattgaggttgataggcactctggaggttgcgat<br>gtgatgttaagctgggggaagactctaag
AT7	ccagttacaagatctaccgcagcccgttaagacatctcattacgcgcacgagtagaaggaaaggcaaatc<br>gaccccgtacaaagaaaaaaacctgtccgagccgctgcactctcctcgtcgagcttaggttcgataaat**C**g<br>**A-TATAACACCACATTTGTTTAACTGCGAATCTGTTG-**<br>**ATTATTGTGT…**	**…TTTCCCGCCTTCAGTTTAAACTATCAGTGTTTGA**T**GGA**<br>**TAT**gaagcaaatttgagccggttgacggatgaggcttctgtagcagaatactgtattgtac<br>tcaaagacgctctagatgggcttgcttgtcttcttagacctgtggcagatctacg

Bold uppercase letters denote nucleotides from T-DNA borders. Flanking genomic sequences are indicated by lowercase letters. Microhomologies between T-DNA and *M. fructicola* genomic sequences are underlined.
